# Data on photosynthetic trend and yield performance of oat (*Avena sativa* L*.*) grown under vertical agrivoltaic system in Sweden

**DOI:** 10.1016/j.dib.2026.112777

**Published:** 2026-04-14

**Authors:** Giovanni Urso, Simone Coluccia, Davide Farruggia, Giuseppe Langella, Mario Licata, Michelina Ruocco, Torsten Hörndahl, Silvia Ma Lu, Arash Khosravi, Pietro Elia Campana

**Affiliations:** a Department of Agricultural, Food and Forest Sciences, University of Palermo, Palermo, Italy; b Greenable S.r.l., Palermo, Italy; c Department of Biological Sciences, University of Naples Federico II, Naples, Italy; d Institute for Sustainable Plant Protection (IPSP), National Research Council of Italy (CNR), Portici, Italy; e Department of Industrial Engineering, University of Naples Federico II, Naples, Italy; f Department of Biosystems and Technology, Swedish University of Agricultural Sciences, Alnarp, Sweden; g Department of Engineering Sciences, Mälardalen University, Västerås, Sweden

**Keywords:** Leaf area index, Biomass yield, Vertical bifacial agrivoltaic, Shading impact, Crop growth

## Abstract

Growing crops under agrivoltaic (AV) systems often exposes them to a modified microclimate, which can affect yield and quality. A field experiment was carried out in 2025 on oat (*Avena sativa* L.) grown within a vertical AV system near Fellingsbro, Sweden. The aim of this study was to assess the growth and yield performance of oat relative to open-field reference conditions. The dataset allows analysis of oat growth by exploiting photosynthetic rate and leaf area index measurements, as well as yield performance at harvest, estimated as total biomass yield (kernel and straw combined). It also aims to explain the impact of vertical AV systems on oat photosynthetic rate, considering different positions within the system with varying shading patterns and sun exposure, and their impact on overall oat yield. These data provide insights into oat growth and yield under vertical AV systems and facilitate further analyses or comparisons across locations or AV system designs. Additionally, the dataset can support the development and validation of crop models under similar conditions and encourage collaborations to promote experimental studies on AV systems.

Specifications Table

**Table:** 

Subject	Earth & Environmental Sciences
Specific subject area	Agriculture and Solar Photovoltaic
Type of data	Tables, graphs, figures, and images. Analysed mean and raw data.
Data collection	Oat data were collected near Fellingsbro (latitude 59°28′05″ N, longitude 15°31′27″ E), Sweden, in 2025. During the growing period, leaf area index was measured using a canopy analyser on 17 and 30 July. Photosynthetic rate was measured using a leaf gas exchange system equipped with a light unit to modify the photosynthetic photon flux density (PPFD) on three dates: 17 July (under ambient light), 30 July (at 550 PPFD), and 14 August (at 1000 PPFD). At harvest, on 3 September, a total of 90 square samples were collected, each covering an area of 0.25 m². The samples were divided into four groups, comprising three groups within vertical AV, each consisting of two subgroups, and one reference group consisting of three subgroups. Each subgroup cantained 10 replicates. Total biomass yield (kernels and straw) in dry matter (kg ha⁻¹) was calculated for all samples. All data were statistically analyzed and are presented here.
Data source location	Mälardalen University, Sweden, is the owner of the data presented in this article. The field trial performed near Fellingsbro, Sweden (latitude 59°28′05″ N, longitude 15°31′27″ E, altitude of 34 m above sea level).
Data accessibility	Repository name: Zenodo Data identification number: 10.5281/zenodo.18196870 Direct URL to data: https://doi.org/10.5281/zenodo.18196870 Instructions for accessing these data: None.
Related research article	None

## 1 Value of the Data

• This dataset provides findings on the effect of a vertical agrivoltaic (AV) system on photosynthetic rate, leaf area index (LAI), and crop yield of oat in Sweden. The dataset contains data comparing three sections within the vertical AV system rows (i.e., West, Middle, and East) to highlight variations in crop growth, and between the AV system and open-field reference conditions. By providing site-specific data, the dataset supports researchers in optimizing AV design and operation, while improving understanding of oat performance and physiological responses under this specific system design in Nordic countries.
• The experimental method used in this study, including photosynthesis and LAI measurements, crop sampling, and biomass yield analysis, is described in detail to allow complete reproducibility of the experiment and data collection at other agrivoltaic sites. Additionally, the experimental protocol can serve as a methodological reference for the comparative evaluation of new AV configurations with alternative crops.
• The dataset provides information on oat productivity under a vertical AV system in Sweden and can be used by agronomists, farmers, and policymakers. It supports informed decision-making and the implementation of measures, such as subsidies or regulations, to address potential yield reductions.
• This dataset serves as a valuable resource for researchers seeking to develop and validate crop models specifically designed for AV systems.

## 2 Background

Agrivoltaics (AV) represents an eco-friendly solution for supporting climate and electrification targets, with the aim of enhancing land use through the co-location of crop production and solar energy conversion [1]. Several studies show that AV systems, through microclimate modifications caused by photovoltaic (PV) panels, facilitate crop growth by reducing water and heat stresses [2,3]. At the same time, crops can increase the effectiveness of PVs through the cooling effect of evapotranspiration in the soil–plant system [4]. However, when considering configuration, geolocation, and crop type, each AV system can create specific microclimatic conditions that warrant deeper investigation. Additionally, this microclimate modification should be carefully monitored to avoid reduced crop performance resulting from decreased photosynthetically active radiation (PAR) [3,5]. Several studies have shown that simulation models can predict crop yields under different microclimatic scenarios in AV systems [3,6,7]. However, accurate predictions require field data for both calibration and validation of these models [3,6,7].

This dataset shows data on oat (*Avena sativa* L.) growth and yield in a vertical AV system in Sweden. Crop growth was monitored through leaf area index (LAI) and photosynthetic rate measurements, while crop performance was assessed through biomass yield calculation. The dataset provides critical insights concerning how oat plants can interact with microclimatic modifications. These data provide AV stakeholders (i.e., researchers, farmers, and policymakers) with the necessary insights to assess land-use feasibility, develop incentives, and make informed crop and system management decisions. Field data can also be used for crop model calibration and validation, helping to predict AV potential and optimize AV performance, thereby ensuring reliable integration of renewable energy with sustainable agriculture [3,8]. To the best of our knowledge, no previous studies have reported photosynthetic rate measurements for oat under AV system conditions; however, it is worthwhile to present a comparison between our methodology and several prior studies focusing on photosynthetic rate measurements under AV systems for different crops (Table 1).

**Table 1:** Comparison of photosynthetic rate measurement methodologies between this study and several other AV studies, including the PPFD levels used for measurements and the time intervals of data collection.

Reference	Crop	Measurement Timing	PPFD Level (µmol m⁻² s⁻¹)
This study	Oat	Hourly (09:00 −15:00)	Ambient light 550 1000
Barron-Gafford et al [9]	Basil Beans Tomato	Hourly (05:00 - 20:00)	Ambient light
Rouini et al [10]	Zucchini	Hourly (07:00 - 18:00)	Ambient Light
Mohammedi et al [11]	Tomato	Once per day (at 12:00)	Ambient light
Hu et al [12]	Soybean	Once per day (between 9:00 - 11:00)	Ambient light
Priya et al [13]	Cranberry	Once per day (between 10:00 - 12:00)	Ambient light
Sturchio et al [14]	Radish Radicchio	Once per day (between 9:30 - 13:00)	Decreasing 1800 to 0 (12 steps)
Zhang et al [15]	Fig	Once per day (time not specified)	1000
Juillion et al [16]	Apple	Once per day (between 10:00 - 12:00)	Increasing 0 to 2000 (7 steps)
Jiang et al [17]	Kiwi	Every two hours (09:00 - 17:00)	Ambient light

## 3 Data Description

**Fig. 1:**
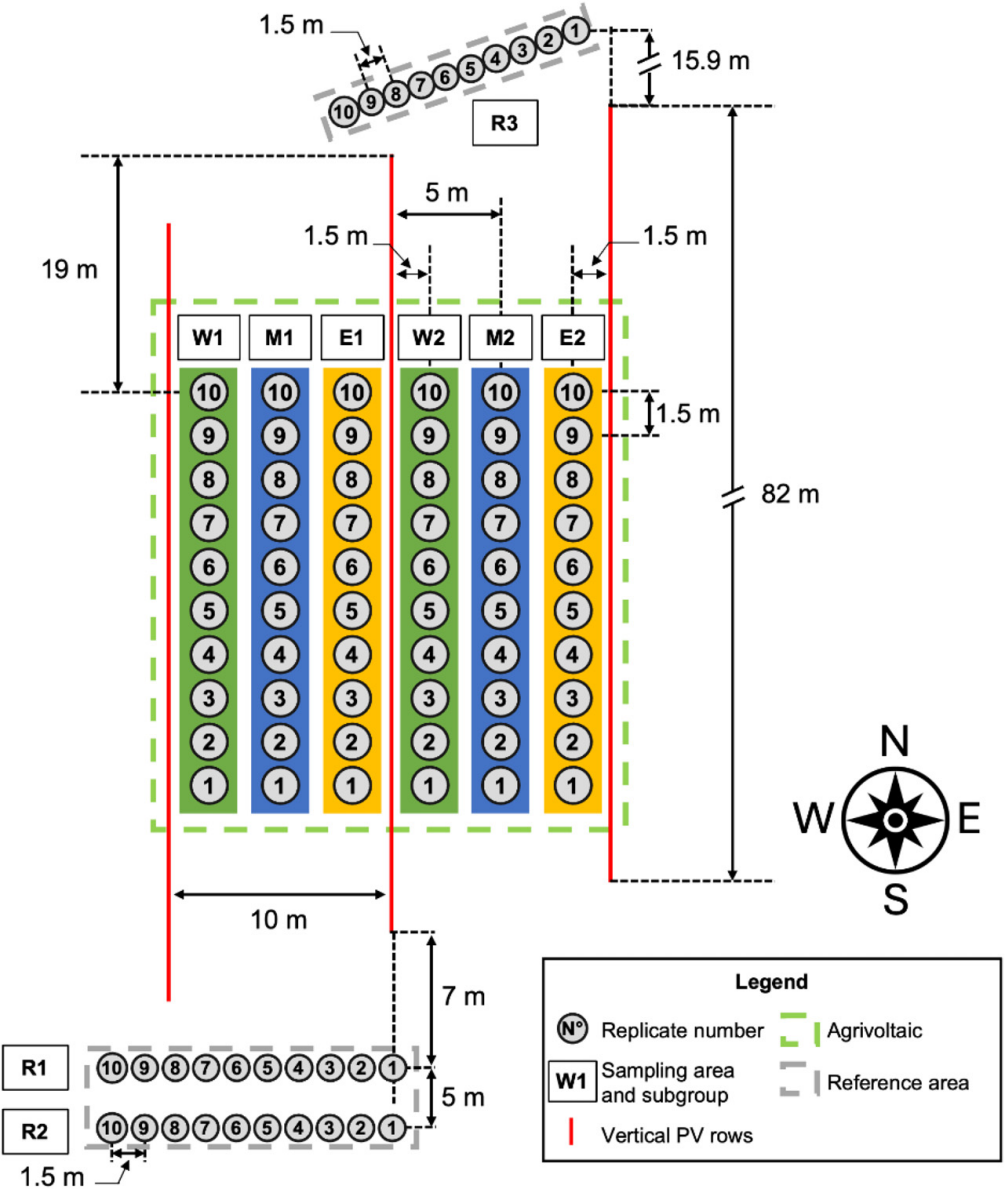
The crop experiment layout (top view) of oat grown in a vertical AV system near Fellingsbro, Sweden.

This study was conducted in 2025 on oat grown near Fellingsbro (latitude 59°28′05″ N, longitude 15°31′27″ E, altitude of 34 m above sea level), Sweden. The dataset includes agronomic measurements and statistical analyses concerning crop growth and yield performance, including leaf area index (LAI, unitless), photosynthetic rate (µmol CO₂ m⁻² s⁻¹), and biomass yield in dry matter (kg ha⁻¹). The statistical analyses were performed considering four distinct groups (W, M, E, R) (Fig. 1).

1- West Section (W): sampling points on the west part between vertical PV rows of the AV system.
2- Middle section (M): sampling points on the middle part between vertical PV rows of the AV system.
3- East section (E): sampling points on the east part between vertical PV rows of the AV system.
4- Reference area (R): sampling points outside the AV system, in full sunlight conditions.

Statistical analysis for total dry matter biomass (Kernel + straw) (kg ha⁻¹) showed significant differences, where the East group had the highest value of average total biomass compared to the other groups (Table 2). West, Middle, and Reference groups produced similar values.

**Table 2:** Statistical analysis of oat for total dry matter biomass yield (kernel + straw, kg ha⁻¹) cultivated near Fellingsbro in 2025 was performed using balanced one-way ANOVA. To compare differences between groups, the Tukey test was used at a 95% confidence level. Different letters indicate significant differences between the groups. No land loss attributable to the PV panel structure has been included in the calculation of total biomass production per hectare for the vertical AV groups (West, Middle, and East).

Groups	Mean	Median	SD	Minimum	Maximum	L.Quantile	U.Quantile
West	6694^b^	6580	875	5360	8520	6010	7230
Middle	7060^b^	6800	952	5000	8640	6500	7790
East	8332^a^	8380	1015	6840	10040	7330	9060
References	6732^b^	6800	1054	4600	9080	5910	7440

Concerning LAI, the first measurement (17 July) showed significant differences between the groups according to ANOVA, with the highest value recorded in the East group (3.4) (Table 3).

**Table 3:** Statistical analysis of oat leaf area index (LAI) cultivated near Fellingsbro for the first measurement date (17 July 2025), performed using a balanced one-way ANOVA. To compare differences between groups, the Tukey test was used at a 95% confidence level. Different letters indicate significant differences between the groups.

Groups	Mean	Median	SD	Minimum	Maximum	L.Quantile	U.Quantile
West	2.7^b^	2.8	0.5	1.7	3.5	2.4	3.0
Middle	2.1^b^	2.1	0.4	1.5	3.0	1.8	2.4
East	3.4^a^	3.2	0.6	2.5	4.3	2.7	4.0
References	2.5^b^	2.3	1.0	1.1	4.9	1.7	3.0

The second measurement (30 July) revealed different results compared to the first one, with the West group showing significantly higher values than the other groups (4.5) (Table 4).

**Table 4:** Statistical analysis of oat leaf area index (LAI) cultivated near Fellingsbro for the second measurement date (30 July 2025), performed using a balanced one-way ANOVA. To compare differences between groups, the Tukey test was used at a 95% confidence level. Different letters indicate significant differences between the groups.

Groups	Mean	SD	Median	Minimum	Maximum	L.Quantile	U.Quantile
West	4.5^a^	0.4	4.4	3.9	5.3	4.2	4.8
Middle	3.5^bc^	0.7	3.6	1.9	4.6	3.1	3.9
East	3.9^b^	0.5	4.0	3.0	4.6	3.4	4.2
References	3.1^c^	0.9	2.9	1.5	4.9	2.5	3.6

**Fig. 2:**
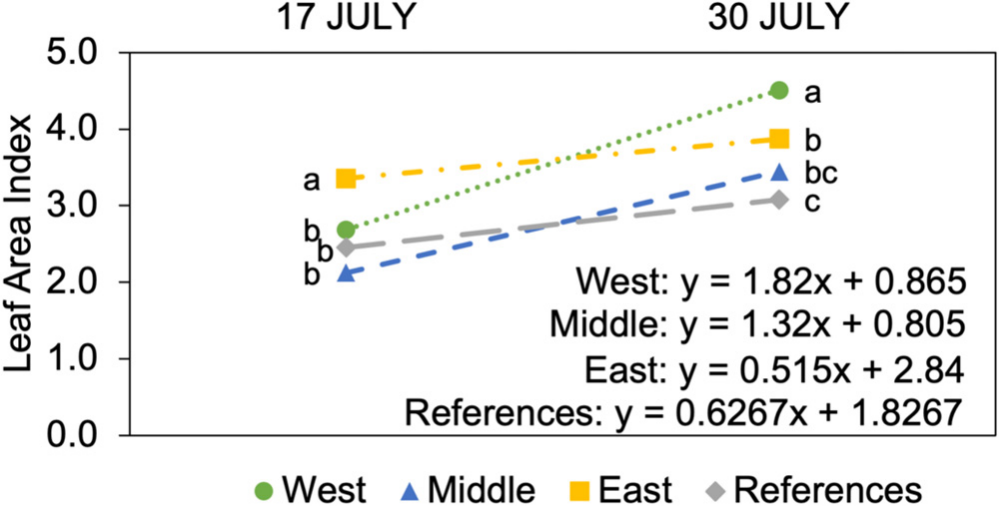
Trend of oat leaf area index (LAI) development across sampling groups on two measurement dates (17 and 30 July 2025). A balanced one-way ANOVA was employed, and differences between groups were compared using the Tukey test at a 95% confidence level. Different letters indicate significant differences.

Across both LAI measurements, the West group showed a faster growth rate than the other groups (Fig. 2).

**Fig. 3:**
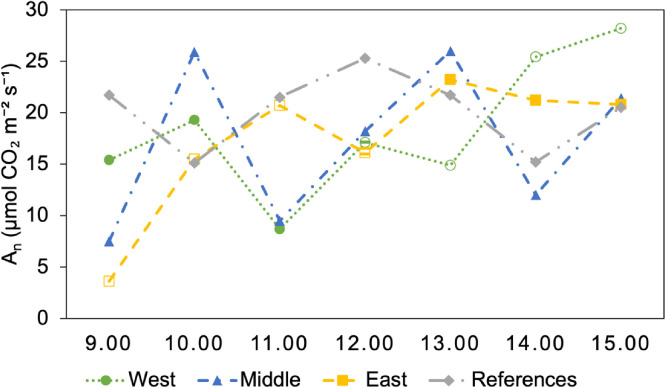
Diurnal photosynthetic carbon assimilation trend over time for one plant in four groups, measured hourly on 17 July 2025. Filled symbols indicate the time points when the plants are exposed to direct sunlight, whereas empty symbols represent the periods when the plants are shaded by the PV panels.

**Fig. 4:**
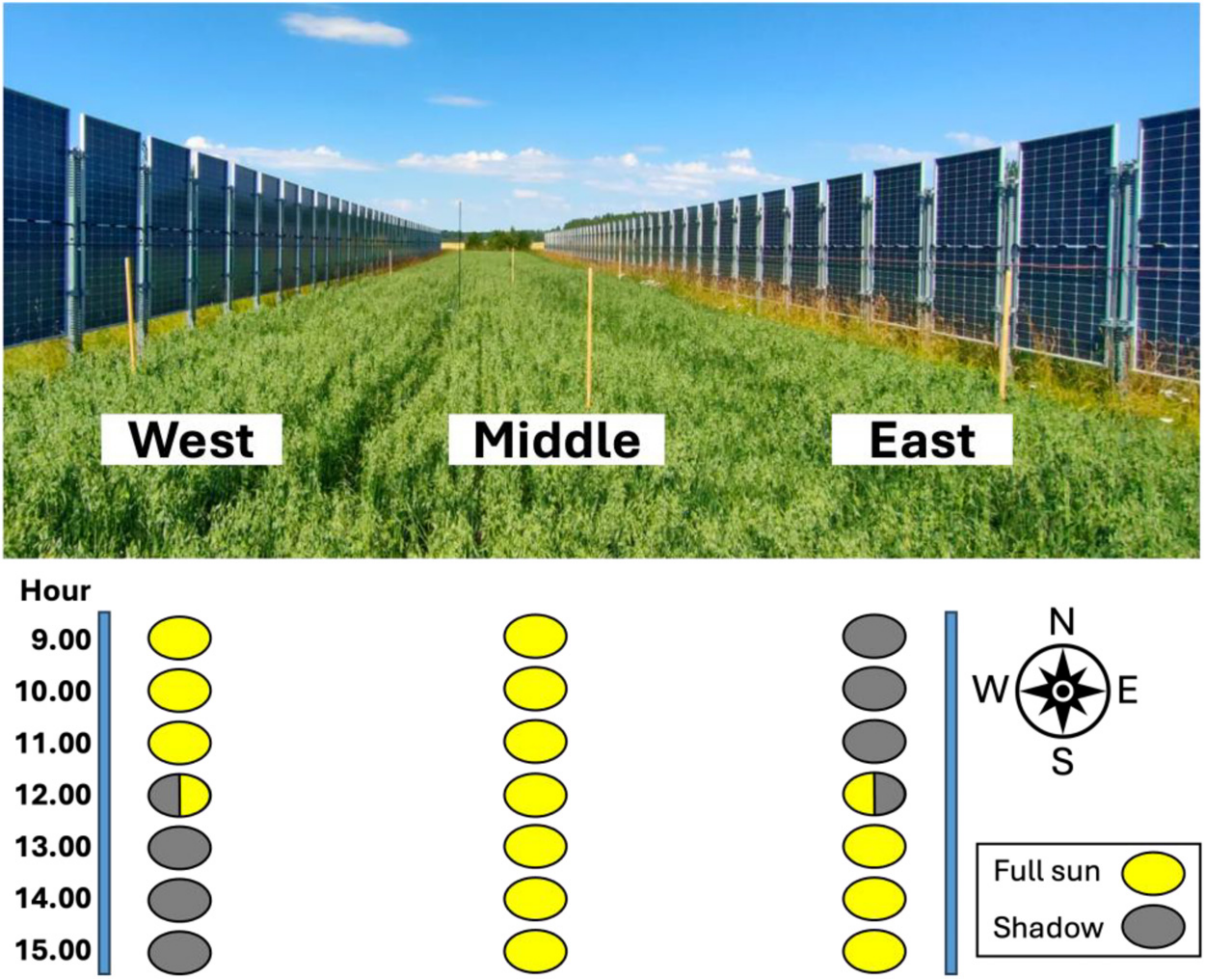
Photo of the vertical AV system taken on 17 July 2025 at 12:00, showing the experimental groups under the West, Middle, and East sections of the solar panels. Coloured ovals at the bottom indicate sun/shade patterns across the groups from 09:00 to 15:00 local time (CEST), with yellow representing full sunlight and grey shaded periods.

Fig. 3 shows the diurnal photosynthetic carbon assimilation trend (A_n_) under ambient light conditions (Fig. 4) (i.e., photosynthetic photon flux density, PPFD, was not modified) on 17 July, between 09:00 and 15:00, for all four groups. There was no single pattern that explained all groups. The References group reached its maximum assimilation at noon (12:00); however, the other groups reached their peaks in the afternoon at different times.

**Fig. 5:**
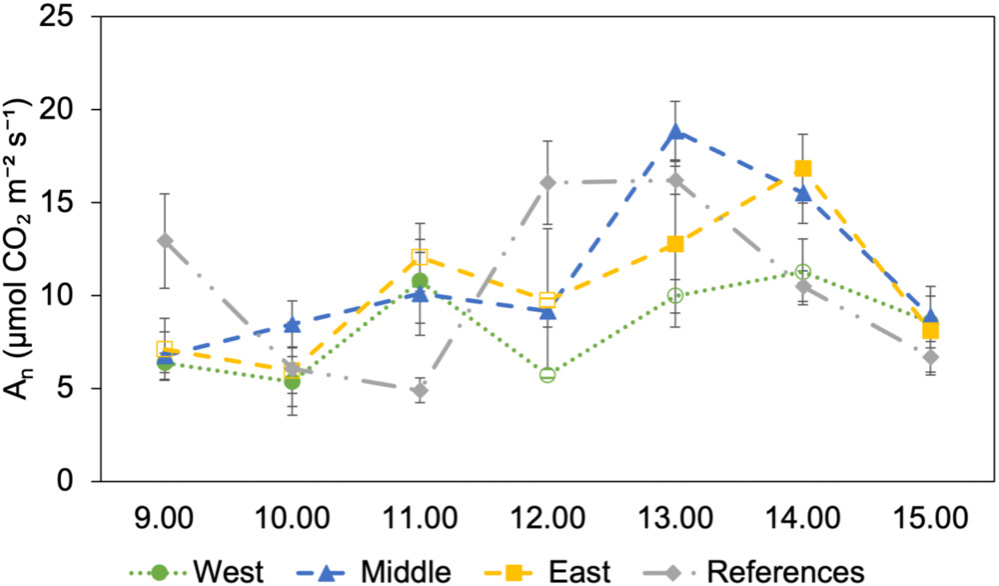
Diurnal Photosynthetic Carbon Assimilation Trend at PPFD 550 µmol m⁻² s⁻¹ for Four Groups, Measured Hourly on 30 July 2025. Each Data Point Represents the Average of Three Measurements, and bars represent standard error (check Fig. 6 for individual measurement). Filled symbols indicate the time points when the plants are exposed to direct sunlight, whereas empty symbols represent the periods when the plants are shaded by the PV panels.

Fig. 5 shows the diurnal trend in photosynthetic carbon assimilation at a PPFD of 550 µmol m⁻² s⁻¹ on 30 July, between 09:00 and 15:00, for all four groups. Each data point represents the average of three measurements. The trends of all other groups were almost similar. The West group showed values ranging from 5.4 to 11.3 µmol CO_2_ m⁻² s⁻¹. The Middle group revealed values from 6.8 to 18.9 µmol CO_2_ m⁻² s⁻¹. The East group recorded values between 6.0 and 16.8 µmol CO_2_ m⁻² s⁻¹. Finally, the References group exhibited values from 4.9 to 16.2 µmol CO_2_ m⁻² s⁻¹.

**Fig. 6:**
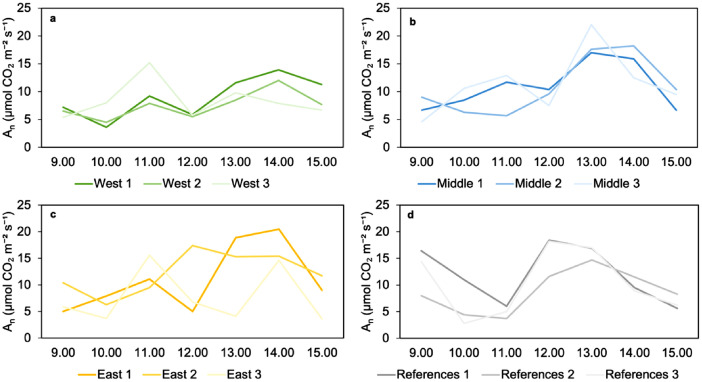
Diurnal photosynthetic carbon assimilation at PPFD 550 µmol m⁻² s⁻¹ for the four groups (a: West, b: Middle, c: East, d: References) on 30 July 2025.

Fig. 6 shows individual measurements at a PPFD of 550 µmol m⁻² s⁻¹ on 30 July, for each group. All three measurements within each group showed the same pattern, except for the East group. However, no consistent trend can be observed when comparing the different groups (Fig. 6).

**Fig. 7:**
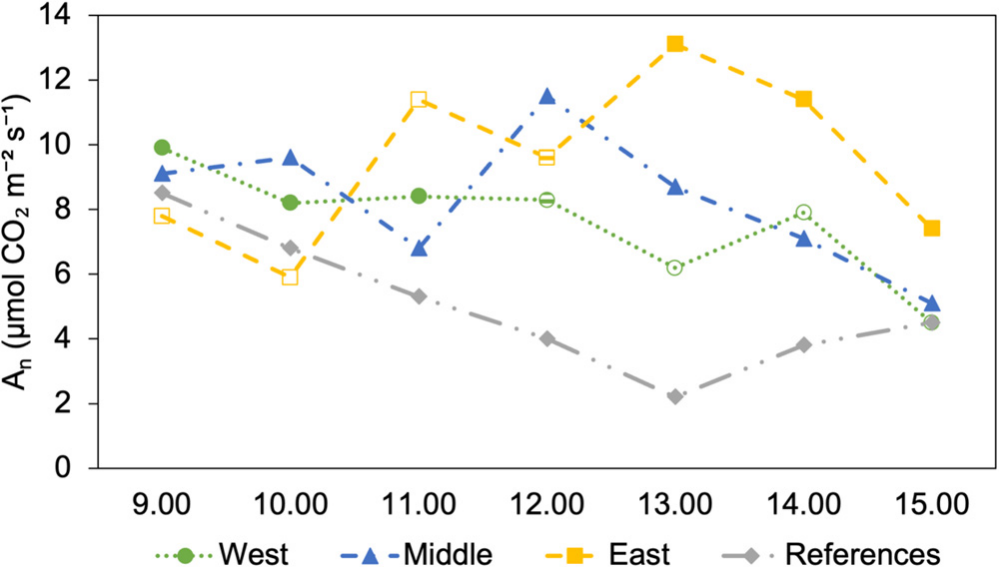
Diurnal photosynthetic carbon assimilation trend at PPFD 1000 µmol m⁻² s⁻¹ for four groups, measured hourly on 14 August 2025. Filled symbols indicate the time points when the plants are exposed to direct sunlight, whereas empty symbols represent the periods when the plants are shaded by the PV panels.

Fig. 7 shows the diurnal photosynthetic carbon assimilation trend at a PPFD of 1000 µmol m⁻² s⁻¹ on 14 August, between 09:00 and 15:00, for all four groups. There was no similar trend among groups.

## 4 Experimental Design, Materials and Methods

### 4.1 Experimental layout and study area

The experiment was conducted in 2025 at the vertical AV solar park near Fellingsbro, Sweden (latitude 59°28′05″ N, longitude 15°31′27″ E, altitude of 34 m above sea level). The AV system covers approximately 1 ha and has an installed capacity of 635.58 kW_p_. It uses a fixed structure installed with the pile drilling method and consists of twenty rows of vertically mounted bifacial PV modules (Longi Solar, 540 W_p_, LR5–72HBD 540 M) with a row spacing of 10 m and a north–south panel orientation (Fig. 4). The modules are mounted in portrait orientation at a height of 2.85 m, positioned 0.6 m above the ground. On 15 May 2025, oat (*Avena sativa* L., variety EOS) was sown under a vertical AV system at a seed rate of 250 kg ha⁻¹, in combination with the application of 350 kg ha⁻¹ of N-P-K fertilizer (24–4–5). The crop was managed under rainfed conditions and harvested on 3 September 2025.

To assess differences among samples, four experimental groups with replicates were established (Fig. 1). The area inside the vertical AV was divided into three sections: West (W), Middle (M), and East (E). Each section consisted of two subgroups: W1 and W2 in the western position, M1 and M2 in the central position, and E1 and E2 in the eastern position. Each subgroup contained ten replicates.

The reference areas consisted of three subgroups, R1, R2, and R3, each containing ten replicates. The W, M, and E subdivisions within the vertical AV system were designed to permit a detailed evaluation of growth dynamics in different locations within the AV system rows. This arrangement enabled assessment of the effects of variations in light intensity and shading on plant development. The reference block served as the control, exposed to full sunlight.

### 4.2 Leaf area index

During the trials, vegetation cover was assessed by measuring LAI in two events, 15 days apart, during the growing season (17 and 30 July). Measurements were taken using the SunScan system (SS1 model, Delta-T Devices Ltd., Cambridge, United Kingdom). An ellipsoidal leaf angle distribution parameter (ELADP) value of 0.9 was applied for all measurements. Data from the W and M sections of the block were collected in the morning, whereas measurements from the E section were performed in the afternoon without shading from the vertical AV structure to avoid interference with the recorded parameters. A total of ninety samples were measured on each measurement date.

### 4.3 Photosynthetic rate

To obtain a clear understanding of the photosynthetic trend of oat plants grown under the vertical AV system compared with the open field, daily diurnal measurements were performed at 1-hour intervals (09:00, 10:00, 11:00, 12:00, 13:00, 14:00, and 15:00 local time (CEST)). Measurements were taken using a portable photosynthesis system (TARGAS-1, PP Systems, Massachusetts, USA) equipped with a light unit to modify the photosynthetic photon flux density (PPFD). Measurements were planned out in three events: 17 July, without modification of PPFD (under ambient light condition); 30 July, with PPFD set to 550 µmol m⁻² s⁻¹; and 14 August, with PPFD set to 1000 µmol m⁻² s⁻¹. On the first and third measurement dates (17 July and 14 August), one representative plant per group (W, M, E, R) was marked, and measurements were performed on it. On the second measurement date (30 July), to capture potential variations in photosynthetic rate within each group, three representative plants per group were marked and measured. At the end of each measurement day, the selected plants were removed from the soil and photographed to document its physiological stage. Physiological data were identified according to the BBCH scale [18] and are presented in Table 5.

**Table 5:** Physiological stages of oat plants (*Avena sativa* L., variety EOS) at each measurement date, determined according to the BBCH scale [18]. Measurements are related to plants grown under the vertical AV system (W, M, E) and the open-field reference (R). BBCH stages are as follows: 7.9 – watery ripening of the caryopsis, seeds morphologically complete but still high in moisture; 8.3 – beginning of seed maturation, onset of senescence, initial dry matter accumulation, first signs of hardening, and straw yellowing; 8.9 – full maturation or nearly complete seed hardening.

Date	West	Middle	East	References
17 July	7.9	7.9	7.9	8
30 July	8.3	8.3	8.3	8.3
14 August	8.9	8.9	8.3	8.9

### 4.4 Crop sampling

**Fig. 8:**
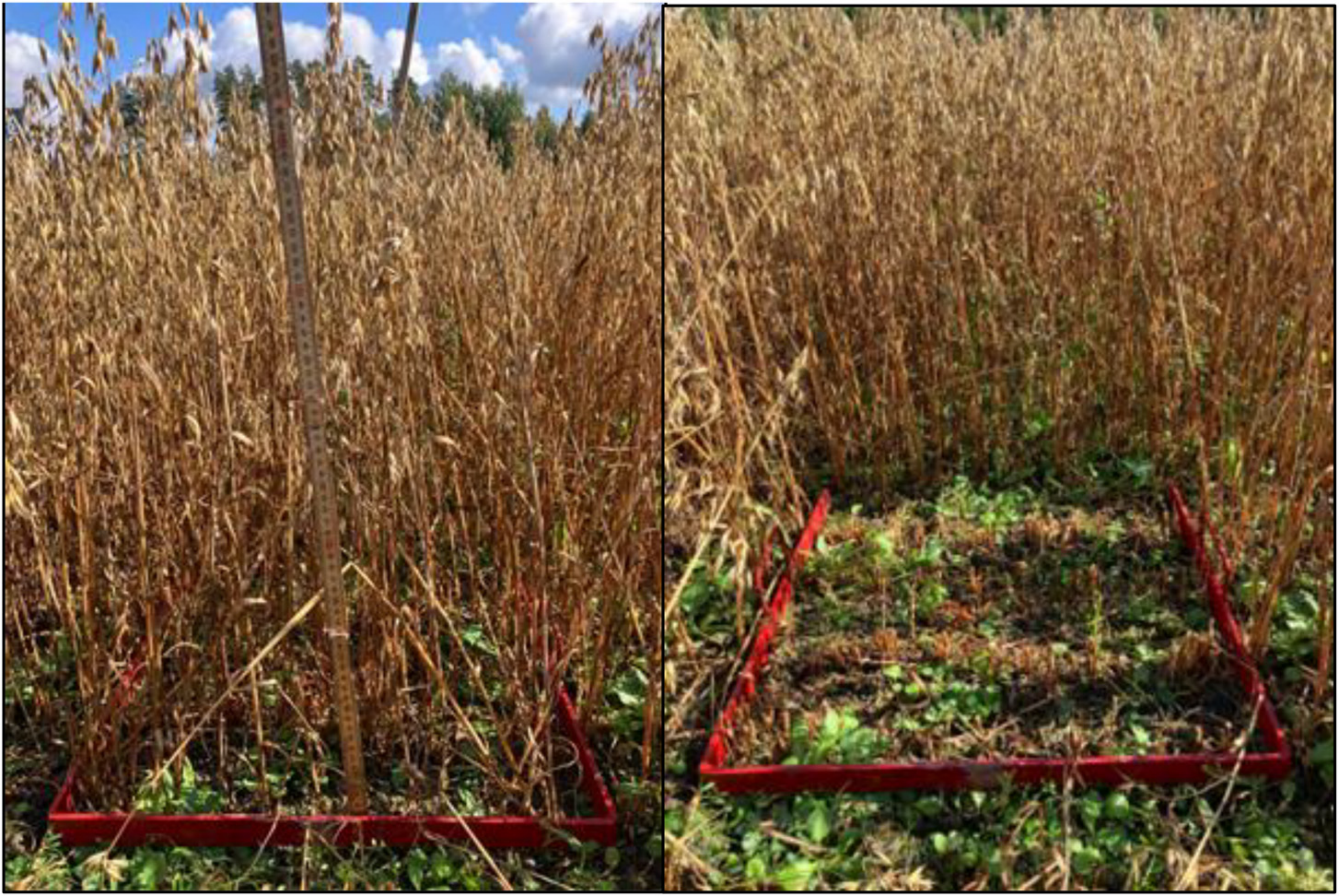
Custom-made rectangular sampling frame (0.5 × 0.5 m) used for oat harvest. Left: frame positioned in the field before sampling, showing standing crop. Right: frame after manual harvesting, illustrating the area and remaining stubble.

Sample collection was carried out on 3 September 2025, when oat had matured. A custom-made rectangular frame (Fig. 8) with dimensions of 0.5 × 0.5 m was used to define a 0.25 m² area, and the crop within this area was manually harvested using a cordless grass shear (Model UM600D, Makita Corporation, Anjo, Aichi, Japan) from 5 cm above the ground. 10 samples were recorded per subgroups, for a total of ninety. Collected samples were carefully cleaned of all weeds, and their fresh weight was measured in the field using an accurate scale (Wellish, model YP20002, with an accuracy of 0.01 g). The harvested samples were taken to the laboratory and dried in an oven at 60 °C for 72 h. After drying, the dry matter content of both kernels and straw was determined by weighing.

### 4.5 Statistical data analysis

Prior to statistical analysis, data were tested for normality using the Ryan–Joiner test and for homogeneity of variances using the Anderson–Darling test. Data were then analyzed using one-way ANOVA with Minitab Statistical Software (version 22.2.1), and significant differences between groups were assessed using Tukey’s HSD test at a 95% confidence level.

## Limitations

It is well known that climate variability greatly affects crop growth and yield in agroecosystems. In the AV environment, local variations in air temperature and rainfall due to climate change create conditions that can modify crop productivity, introduce uncertainty, and complicate management strategies [19]. According to data from the precipitation station of the Swedish Meteorological and Hydrological Institute (SMHI) in Örebro, the closest to the experimental facility, the site recorded average monthly temperatures for May to August 2025 that were close to normal. This is because only May had an average temperature deviating by more than ±0.6 °C from the average monthly value for the period 1990–2020. Even though total precipitation for the same period was close to normal, rainfall in June and July exceeded the 1990–2020 average by 28% and 43%, respectively. It is evident that mild or extreme weather can differently affect the resilience of the same crop under AV panels, providing diverse performance in terms of yield and quality. In this study, one-year trials were carried out; however, crop performance can vary year to year, and therefore one season is not able to explain long-term variability. However, short-term data are also valuable for identifying preliminary trends and understanding immediate crop responses to AV configurations. Long-term datasets are crucial to assessing the stability and reproducibility of the results. It is worth noting that publishing short-term datasets is of strategic importance to companies and stakeholders currently active in or interested in the AV sector. Short-term studies can provide early insights to drive technological innovation and investment choices, while long-term data offer the scientific robustness needed to support large-scale applications, policy development, and the sustainable increase of AV systems. Another limitation of this study is the reduced number of samples and replicates. This challenge, however, is common in agronomic trials involving AV systems, where physical space and technical constraints, especially in non-commercial setups, can greatly limit the replication size [8,19,20]. Moreover, due to technical constraints, only a limited number of crops can be tested by exploiting an appropriate experimental design.

## Ethics Statement

The dataset collected in this study did not involve animals, humans, or any data collected from social media platforms.

## Credit Author Statement

**Giovanni Urso:** Writing – original draft, Formal analysis, Methodology, Investigation. **Simone Coluccia:** Writing – review & editing, Formal analysis. **Davide Farruggia**: Writing – review & editing, Visualization. **Giuseppe Langella:** Writing – review & editing. **Mario Licata:** Writing – review & editing, Formal analysis. **Michelina Ruocco** Writing – review & editing. **Torsten Hörndahl:** Writing – review & editing. **Silvia Ma Lu:** Writing – review & editing, Visualization. **Arash Khosravi:** Writing – original draft, Formal analysis, Methodology, Investigation, Conceptualization, Supervision. **Pietro Elia Campana:** Writing – review & editing, Resources, Funding acquisition, Methodology, Conceptualization, Supervision.

## Data Availability

ZenodoData on photosynthetic trend and yield performance of oat (Avena sativa L.) grown under vertical agrivoltaic system in Sweden (Original data).
